# History of Foundlings in Austria, with Especial Reference to Their Condition in Illyria

**Published:** 1846-10

**Authors:** 


### Art. V.

*Geschichte der Findlinge in Oesterreich mit besonderer Rücksicht auf ihre Verhältnisse in Illyrien.* Von Dr. Raimund Melzer, K.k. Director der Staats- und Local-Wohlthätigkeits-Anstalten zu Laibach.—*Leipzig,* 1846.

*History of Foundlings in Austria, with especial Reference to their Condition in Illyria.* By Dr. Raimund Melzer, Imperial Director of the State and Local Charitable Institutions at Laybach.—*Leipsic,* 1846. 8vo.

The author of this able and learned work is unusually well qualified for the task he has set himself. Trained in the service as well as in the superintendence of the commission for inquiring into the state of the poorhouses and houses of correction, then acting in the capacity of professor of midwifery and physician to the lying-in and foundling hospitals, and, lastly, appointed to the responsible post of director to the state and local charitable institutions, he has been diligently employed, during ten years, in the performance of duties which have familiarized him with the causes and effects of poverty. It was natural that the institutions with which he was most intimately connected, first as physician, and subsequently as director, should engage the greater share of his attention. Accordingly, it scarcely required the stimulus of the example of the prize essays of MM. Terme and Monfalcon, of which the reader will find elaborate notice in the Twenty-sixth Number of our Review, to urge him to the laborious inquiries which have issued in the work before us—a work for which his habitual occupations, and his access to public documents, peculiarly fitted him.

His mental qualifications for his task will appear of no mean order to any one who will take the pains to peruse his Introduction, in which he shows that he has not been a careless observer of poverty and the poor, and that the great and perplexing alternations which have beset all national efforts for the relief of indigence have not escaped him. These alternations have rarely been better expressed than in the following passage:

"Poverty has the great misfortune that it borders closely on vice, and easily passes into it. Nay, we may call vice a moral poverty. Two steps further, and the pauper becomes the criminal. If charity is not circumspect, it will be deceived and cheated by vice or crime, and innocent poverty will be robbed of the assistance which was intended for it. How easily this may happen may be inferred from the familiar fact that natural benevolence is easy of belief, and from the mistakes which philanthropic persons are continually making. As often as poverty is mixed up with vice it is in a state of resistance against law, for vice is in its very nature lawless. This circumstance is the source of many evils with which even the best administration of Foundling Hospitals has to contend, and is the reason why several governments exclude those establishments from the lists of charitable institutions, and esteem them a greater evil than the existence of those whom they are intended to assist."

Our author shall state in his own words the case of foundling hospitals, and shall exhibit the way in which the charity which presided at their foundation has been and is deceived and cheated:

"The Christian pity," he says, "which called these institutions into being, went so far in its anxious care for mother and child as in their reception to make itself blind. These institutions had not existed long before they became the dupes of vice. They were established to preserve purity of morals, and they soon began to undermine them. The stricter the principle of secrecy was preserved, and the further it was carried, the more shamelessly was it abused. Parents, bound by the ties of marriage, forgot their most sacred duties, and relieved themselves of children whom they regarded as heavy burdens; and they did this because the regulations of the institution tempted them to it—because they saw themselves thereby exculpated from a punishable offence, and even justified in their unnatural conduct. The foundling hospitals were crowded with applicants, and the expense increased with their increasing numbers till the evil reached an alarming height. To prevent this monstrous outlay from increasing, and, if possible, to effect a permanent diminution of expense, without endangering the original humane aim of the institution, formed the problem; the solution of which became the anxious wish of the government and of every friend of humanity."

And is not this the history of all charities without exception, and especially of all so-called national charities? Let us suppose a large town, if such a supposition be reasonable, in which no such thing as a beggar is to be found, but it is known that a large number of its inhabitants are poor, many in want, and some few, perchance, starving. A benevolent person, aware of these facts, proclaims them, and appeals to his fellow-citizens in behalf of the sufferers, insisting that they ought to carry purses in their hands, and invite the destitute to apply to them for alms as they walk the streets. What would be the consequence? Why, what other result could follow but that which has followed from the habit of dropping halfpence into the hands of street beggars—the creation of a band of masqueraders, who make mendicancy their profession, and come to think it to the full as honest as working with their hands, and somewhat safer than stealing, but who are much more likely to forget the danger of petty larceny than to be reminded of the safety and respectability of honest labour. And what is the total result of this misguided charity? This: to draw off, by the constant dropping of halfpence and pence into idle and unworthy hands, the fund which was destined to the support of honest labour, and which, so applied, would have rescued a greater number of deserving people from want, and saved more victims of threatened starvation than all the idle almsgiving in the world. It would probably have prevented. and most certainly have contributed to prevent, that very distress which brought the idle habit of street-almsgiving into existence.

At length the public, though slow to learn, discovers the error into which it has been led, and street-begging begins to be discouraged. Naked feet, bandages, and tattered fancy dresses are at a discount, the Mendicity Society is in favour, and charity takes a new direction. She has been imposed upon in the street; she will try the houses. There, at least, she must be safe. She can inquire into the particulars of every case of destitution and suffering, and her purse-strings shall not be drawn till she has satisfied herself that her eyes are not deceived, nor her heart melted by the tricks of the theatre. Poor dupe! Broken chairs, tattered and scanty bedclothes, and all the marks of domestic poverty and wretchedness, have replaced the rags and bandages of the street, and the affected piety of the home hypocrite and masquerader, the whine of the street-beggar. And here, too, the funds which might have ministered to the support of honest industry, and the relief of real want, are diverted from their proper channel by the treacherous arts of the house-beggar. Take one other case. This nation piques itself upon its poor-law, and on its generous determination that no man in the whole length and breadth of England shall be allowed to starve. Good. Britannia looks well in the attire of my Lady Bountiful. But is she quite sure that she does more substantial good by her annual expenditure of millions—that is to say, more good in proportion to the outlay—than the patron of street-beggars or home-impostors? Is out-door relief more free from imposition than street-almsgiving, or the union system much more free from abuse than the district visiting societies? Are not the millions expended every year as certainly drawn from the fund which would support honest industry and *prevent* destitution? and is the total good effected in any degree proportionate to the total expenditure? A fact was recently mentioned in the course of a debate in the House of Commons, which, strange to say, seems to have passed unnoticed by those who have the eye of a lynx for any abuse of the poor-laws, provided it be only traceable to the faults of their administrators. As many as four thousand pensioners were distinctly stated to have squandered their pensions in drink, and then to have entered the workhouse. Of course they did. What better could be expected of a certain considerable portion of society than that they should avail themselves of the houses so kindly opened for the encouragement of idleness, intemperance, and profligacy. Then what an instrument of rural tyranny that same workhouse system is! How convenient the threat that if wages, brought to the level of starvation, be not accepted, there is the workhouse hard at hand, where the honest labourer shall be introduced to all the idleness and profligacy of his neighbourhood, and supported (cruel mockery!) by the very funds which ought to have contributed to pay him a fair day's wages for a fair day's work.

We have been provoked to say more than our readers might expect to find in a Medical Review on this great question of so-called charity, individual and national, and to throw out these evidences of a scepticism as to the real good effected by its means, by our author's unjust censure of a man who, whatever his faults as a reasoner, cannot justly be accused of any want of humanity, in spite of the severe and, in some respects, well merited strictures of Sadler,—we mean Malthus.

The theory of Malthus was, doubtless, a false theory, and his fears of an increasing population idle and visionary; but his opposition to the poor-laws, and his assertion that no man has an abstract right to support, whether he will or will not work, may be, in reality, in its necessary results, far more humane than the easy and good-natured theory which it opposes. The question really is, whether the funds devoted to charity effect more in the way of cure than these same funds, employed in the support of labour, would accomplish in the way of prevention. Malthus may have been wrong in upholding the latter alternative; but it does not follow that his error, if it be one, was the offspring of an unfeeling disposition.

With this short protest against our author's disrespectful notice of the tenets of Malthus, and without stopping to qualify our own broad strictures on the evils of many of our modern charitable institutions, we proceed to a more intimate examination of the question of Foundling Hospitals. Considered in the light of charities, they are peculiarly open to the abuses which cling to all ill-considered plans for the relief of suffering and destitution; and it happens, fortunately, that the shrewd sense and right feeling of Englishmen have, very generally, condemned them; though with the narrow and exclusive views which, unfortunately, characterize highly practical nations as well as highly practical individuals, the merited censure they have received has not been extended to other so-called charities open to similar, if not greater, abuses. At first sight it would seem an unmixed good, as well as an obvious Christian duty to succour and support such children as may be found abandoned by their unnatural parents; but this duty must have some qualifications, and this good some limits. What virtue or what blessing has not? Where, then, are these qualifications and limitations to be found? The answer is easy: when the practice of a duty, or what appears to be such, entails more evil and more suffering than it relieves, it is obvious that we have been acting under a mistake, and that we must abandon or modify our course of procedure. The point at which the evil predominates over the good is evidently the limit which we ought not to pass beyond. In the case under consideration, the object we have in view is the preservation of human life.

This just and sacred object we, undoubtedly, accomplish so long as in our reception of the foundling we hold out no encouragement to illicit intercourse, or the multiplication of deserted children. But no sooner do the measures which we adopt, whether as individuals or communities, act as an advertisement of our charitable intentions and actions, than deserted children begin to increase till, at length, they are equal to the utmost demand which our philanthropy has created. Let us take the extreme case, that children of the tenderest age are received at all hours of the day and night, without question or limitation, as was the case, till recently, in France, under the liberal system of *tours;* and it follows, inevitably, that we are the indirect means of destroying more lives than would have been lost, had every foundling been suffered to perish in the streets. For we contrive, at one and the same time, to hold out an encouragement to illicit intercourse, and to the barbarous desertion of legitimate children; and by bringing so many children together under circumstances so unfavorable to infant life, we are the instruments of a more wholesale destruction of life than could have happened, had the comparatively few deserted children been left to the casual care of individual philanthropists, or to the fate to which their unnatural parents had abandoned them.

The suppression of the *tours* was, doubtless, a great improvement on the old system ; but even with this improvement it admits of grave doubt, whether the encouragement held out to illicit intercourse, on the one hand, and the mortality incident to the best regulated foundling hospitals, on the other, do not lead to a larger number of deaths than would have happened had the matter been left to take its own course.

But we have entered quite as much at length into this subject as our limits will allow ; we are bound to proceed to the further notice of the historical and statistical details prepared for us by the industry and research of our author.

The history of the establishment of foundling hospitals in Austria closely resembles that of France. A citizen of Laybach, one Peter Bellach, in the year 1041, devoted the whole of his property to the establishment of an orphan-institution, which was also to receive foundlings, and to train them to a trade. The example of Laybach was soon followed by other towns, and, at length, in Austria as in France, the foundling hospitals fell under the superintendence of government.

The history of legislation on this important subject forms the subject-matter of 170 pages, which we pass over, as it scarcely admits of being rendered interesting to the medical reader; all that can be of any importance to him will be found in the following notice of the statistics of the subject, which occupies the remainder of the volume.

*The number of foundlings,* in the several foundling hospitals of the Austrian dominions, has increased, during the twenty years from 1820 to 1840 in an arithmetical progression, and has doubled within that period. The average yearly number amounted to 49,317. In the year 1839 the population of Austria, exclusive of Hungary and its dependencies, was 23,389,959 ; the number of foundlings 59,026, being 1 in 396. The total in the twenty years amounted to 986,345, or little short of one million! In the years 1833-40, the total number of new-comers amounted to 128,658, or an average of 16,082. The numbers received in 1840, bore to those received in 1833 the proportion of 116 to 100; Gallicia is excluded from this calculation.

In Laybach the increase of foundlings has been very considerable. In the year 1763 one foundling was admitted; in 1842, eighty years later, the number amounted to 201 ; in the first twenty years there were 77 admissions, in the second twenty years 962, in the third 1439, and in the fourth 3275. The increase of females received into the lying-in institution in the same town was also considerable and progressive, though not so great as in the case of foundlings; the out-patients fluctuated, but showed, on the whole, a tendency to increase.

What is the cause of the increasing number of foundlings? is a question of considerable interest to which our author devotes a short chapter. The number of foundlings, as we might anticipate, is chiefly recruited from illegitimate births, which, as a general rule, increase with advancing wealth and civilization. In accordance with this acknowledged principle, it is found that the number of foundlings is least in the poorest districts; poverty, therefore, is not the cause of the abandonment of children. This position is abundantly proved by an appeal to facts. The most prosperous years are shown to be those in which the number of foundlings is greatest, while years of scarcity and war have often been marked by a decrease in number. Returning prosperity, also, is marked by an increase of foundlings, and the periods of the year in which there is the greatest distress, the winter months, are characterized by no marked influx of deserted children: thus, in Laybach, the number of foundlings in July and February was within an unit of the same. In Krain the number of deserted children was greatest in the month of May; March and April come next in order, then August. In the foundling hospital of Vienna, the months stood in respect of births, in the following order, beginning with that in which the number of foundlings was the greatest,—February, March, May, January, April, October, December, September, June, November, August, July; but the greater number of foundlings were, at Vienna also, received in summer.

It would appear, then, that vice is more influential in increasing the number of foundlings than poverty, and that this species of vice at least increases with wealth and civilization.

The next question which Dr. Melzer examines is the proportion of foundlings born in wedlock and deserted by their parents. We were scarcely prepared for so startling a result as that which he has deduced from the records of the foundling hospitals of some of the Italian cities. During the period of ten years, from 1830 to 1840, there were received into the *tours* of the hospital at Milan 26,147 foundlings, of which 6610, or 25 per cent., were legitimate; in Vienna, for the same period, there were only 111 in 3424, and in Brescia, 1744 in 20,149. In Protestant countries where there are no foundling hospitals and no *tours*, these children would not have been deserted by their parents; the Roman Catholic system, as our author observes, must, therefore, bear the blame of this increase of foundlings.

Another abuse of the foundling system is the abandonment of children by mothers, who offer themselves, immediately after, as nurses in the hope of being paid for tending their own children; this abuse was pointed out, as occurring in the French hospitals, in the review to which we have already referred.

The mortality in childbed and that of foundlings next engages the attention of our author. The mortality in childbed at the lying-in institution at Laybach, has been very low compared with that of many other similar establishments in Austria. While it has been 6 per cent. in Vienna, 5 in Milan, 3 in Lemberg, 2 in Prague, Linz, and Venice, it was less than 1 per cent. in the Tyrol, and 80 in the 1000 at Laybach. This high mortality of Vienna is attributed, and with apparent justice, to overcrowding; and our author strongly recommends increased accommodation, quoting, for this purpose, the dictum of Dr. Ferguson, "a lying-in hospital should consist either of a series of cottages, or its spacious wards should contain very few patients."

The mortality of children is very minutely examined, beginning with still-born children; it appears that in the several lying-in institutions of the Austrian Monarchy, they bear to those born alive the following proportions:—Gallicia, 1 in 7; Dalmatia, 1 in 11; Lombardy, 1 in 12; Maritime districts, 1 in 13; Venice, 1 in 16; Upper Austria, 1 in 18; Lower Austria, 1 in 25; Steiermark, 1 in 26; Tyrol, 1 in 27; Bohemia, 1 in 28; Moravian Silesia, 1 in 38; Carinthia, 1 in 129. The average is 1 in 24; the proportion of still-born males to females was 124 : 100. The autumn months were found to be most fatal, then the winter and spring, and the summer least fatal; but to this general rule Vienna forms an exception, the greatest mortality occurring in summer. In the Laybach institution premature births occurred in the proportion of 1 in 44 mature births; of these premature births, the greatest number occurred in the eighth month. Out of 91 premature births 5 (3 boys, and 2 girls) survived, and 86 were born dead. The still-births were, therefore, to those born alive, as 17 to 1; and the proportion of males to females as 139 to 100. The proportion of twin-births, in one of the lying-in institutions, during a period of fifty-one years, was 71 in 4669, or 1 in 66. In France the proportion is 1 in 95; in Germany, 1 in 80; in England, 1 in 92; in Scotland, 1 in 95; in Ireland, 1 in 62. In these twin-births there was one male to three females; 17 twins in the 100 were born dead, and where one was born alive and the other dead, the number of still-born males was, to that of still-born females, as 1 to $2\frac{1}{3}$. The accoucheur will find some interesting details of the relative frequency of natural and unnatural presentations, to which we refer him, while we proceed to the mortality of the inmates of foundling institutions. In a period of fifty-three years there were born in Krain, on an average, 102 foundlings, of whom, 67 attained the age of ten years, and 33 died before that age. In the first twelve years of life the deaths numbered $34\frac{1}{2}$ per cent.; the mortality of the first year was 23 per cent., which fell short of the mortality in the community at large, which amounted to nearly one third. The mortality of male children was to that of females as 6 to 5; but it must be borne in mind that the male children exceeded the females by 5 per cent. The mortality of males, therefore, supposing the numbers of the two sexes to be equal, evidently exceeds that of females.

If we divide the fifty-three years from 1789 to 1841 into two parts, it will be found that the mortality of the first half is less than that of the second; the cause of this difference is not stated; it would probably be found to depend on increasing numbers, without a corresponding increase of accommodation.

The mortality of foundlings in Vienna is minutely examined by the aid of figures. During twenty years (from 1821 to 1841) the mortality ranged from 12 to 23 per cent. In the first ten years the average mortality was lower than in the last, and in the three years, 1839-41, it was much greater than in the three years, 1821-23; the mortality of foundlings in Vienna has, therefore, increased. It appears that in different parts of the Austrian Empire there is considerable difference in the mortality of foundlings; thus, while in Lower Austria it is 18 per cent., in Bohemia 15, and in Steiermark 11, it is only 6 per cent. in Upper Austria and Illyria. This is the mortality of those brought up out of the foundling hospitals.

The mortality of foundlings is still further investigated for all parts of the Austrian dominions. This, as it would interest a small portion only of our readers, we shall pass over, and merely notice such facts as are of general interest.

It has been stated that the mortality of foundlings in Vienna has increased of late years; this is the case also in many other parts of the Austrian dominions, while in about an equal number of districts it has fallen off. The cause of this difference is not stated.

In illustration of the danger attending the exposure of the new-born infant to cold, some interesting facts are stated on the authority of Trevisan. Of 100 children born in Italy during the winter, 66 died during the first month, and only 19 survived their first year; while, on the other hand, of 100 children born during the summer, 83,—of 100 born during the spring, 48,—and of 100 born during the autumn, 58 survived the first year.

Dr. Melzer's work abounds in statistical details, which want of space prevents us from noticing at greater length; we must, therefore, hasten to the concluding chapter of the work, in which the real value of the foundling-hospital system is tested by argument and fact. We have already touched upon this subject, but its importance demands a further notice.

The foundling system is differently ordered in Roman Catholic and Protestant countries. Though the object in both is the same—the preservation of the life of the innocent and helpless child, and the support of public morality,—they aim at accomplishing the object in opposite ways. The Roman Catholic system establishes lying-in and foundling hospitals, forbids any inquiry into the paternity of the foundlings, and loads the state with a heavy expense. The reception of the foundling is either by the *tours*, in which the concealment of the birth is the leading object—this is the Italian system; or the child is received *à bureau ouvert*—this is the French system. These are the two chief varieties of the Roman Catholic system. The Protestant system, on the other hand, pronounces lying-in and foundling hospitals to be an evil, and rejects them altogether; disapproves concealment, makes the mothers answerable for the care of her child, and fines the father in the cost of its support. Where this system prevails illegitimate children are even more numerous than in Roman Catholic countries;* but children are very rarely abandoned. In a financial point of view, of course, this system has a great advantage over that of Roman Catholic countries.

It is impossible that these two systems can be equally valuable; the one must be better than the other; nevertheless, though different in their principles, they show a certain reciprocity in their operation. In the ultimate results of each system we must seek for the proofs of its utility. We must weigh the profit of each system against the moral and pecuniary results, to determine which has the advantage over its rival; but in actual practice we do not find such a relation between the principle and its results as we might have anticipated. To exhibit the real state of the case is the

* The proportion of illegitimate to legitimate births in England is 1 in 12; in France, 1 in 13; in Saxony, 1 in 7; in Würtemberg, 1 in 7·8; in Hesse, 1 in 5·6, in Prussia and Sweden, 1 in 13. (Springer's Austrian Statistics.)

object of political economy; but, in the mean time, let us compare the argument for and against each of these rival systems.

The supporters of the Roman Catholic system shall have the first word. According to them foundling hospitals were among the first-fruits of the divine principle of the love of one's neighbour. Religion, not powerful enough to banish vice, and fearing to stain herself with the blood of the innocent, threw over it the veil of compassion, and led back the unfortunate offender to the bosom of society, out of which the hand of her parents had thrust her. Grief, shame, privation, and despair of being able to preserve the life of her offspring, offer a strong temptation to desertion, child murder, and abortion. To force the father to marry the woman he has betrayed, would be a doubtful good, to oblige him to support his child, is a certain difficulty; but in the asylums which are open to the mother, and in which she finds every needful assistance, she is safely and secretly delivered of her burden, and returns in peace to the world; she escapes the shame and reproval which otherwise await her, conceals her first offence, and can resolve against its repetition. The foundling hospital completes what the lying-in institution had begun; it relieves her from a heavy burden, and prevents her from sinking to a lower point of degradation. The child, whom the mother has consigned to the care of the foundling hospital, is not merely supported, but educated. How many mothers, a prey to shame and destitution, and sorely tempted to the commission of crime, has this system saved from destruction! how many children has it rescued from death* and from the fatal influence of bad example, and preserved to the state as moral and industrious citizens!

If we now turn to the Protestants, they admit the good intentions which led to the establishment of lying-in and foundling hospitals; but they look to their results, and inquire how far the sanguine expectations of their founders have been sealed by experience. These institutions were established to afford the poor destitute mother and child the assistance they so much require; but, however fine in theory, experience says little in their favour. Where such institutions exist, we are constantly meeting with the most melancholy results of such experiments,—men separated from their parents, strangers in their land, and brought up by hireling nurses and attendants. What might once have been a necessity, has long ceased to be such. The public opinion has come to pass a milder sentence on unchastity. By the tenderness which the law has shown to the shame of the mother, shame is at length banished, and with it all fear of consequences. If her poverty be assigned as a reason for our interference, the question arises, is the state bound to undertake the support of all the poor without exception? By doing this, it would step into the place of individual charity; it would give to poverty that which diligence and virtue ought to give it; and, by so doing, would encourage a mean spirit of dependence. The poor ought, it is true, to be supported, but the support offered by the lying-in and foundling hospitals is useless, at the same time that wisdom requires and offers better means. Among the poorer classes there are many married couples who look with envy

* In Carinthia, which has no foundling hospital, 23·57 per cent. of children died under one year of age; in Krain, which has such an institution, only 20·36 perished.

on the illegitimate child and on her offspring, and on the liberal assistance and support which they have received in the lying-in and foundling hospitals. The child is supported at the public expense, and the mother admitted as wet-nurse into some rich or distinguished family. The conviction that, on the whole, the condition of the unmarried female is more tolerable than her own, that her own children can have no better care than that which the foundlings receive, is not far removed from the wish to turn those public institutions to her own use. This wish soon ripens into act, where the institution gives facilities for it. Hence the multitude of legitimate children who are taken out of the *tours*, and which increases as the fact becomes notorious. The mother has taken her legitimate child to the *tour*, the next step is to offer herself, after a few days, at the institution as wet-nurse; when she has the chance of receiving back her own child, and being paid for its support. To what abuses such a system may lead it is unnecessary to point out; the abuses of the system extend until large numbers of females, in a condition to support their own children, avail themselves of these public institutions. To return to the case of the single woman,—the support of her child, which, in the absence of these institutions, must have devolved upon her, might perhaps have saved her from a repetition of her fault; but the relief from these cares tempt her back to her former mode of life, and encourages her in her vicious course; and yet we hear of the moral uses of the foundling hospital, and of its favorable influence on public morals. It is asserted that lying-in and foundling hospitals tend to preserve the lives of children, and to prevent the exposure and murder of the new-born child. Experience, however, has shown how unfounded this statement is; it has proved that there are more exposures and infanticides in countries which have lying-in and foundling hospitals, than in those which are without them; and the same difference is found to obtain in the same countries before and after the establishment of their hospitals. Thus, in Krain, before the opening of the foundling hospital, the annual exposures amounted to $3\frac{1}{2}\frac{5}{1}$ per cent.; after the opening of the institution $4\frac{1}{5}$ per cent. That criminal abortions and infanticides are not diminished by foundling hospitals, is also proved by tables to which we are obliged to refer the reader; neither is the assertion that foundling hospitals preserve the lives of children better founded. Facts are opposed to it; but for these also we must refer to our author.

The result of the careful inquiry into which Dr. Melzer has entered, seems to be unfavorable to the pretensions of what he designates as the Catholic system; and we are not the less inclined to accept the conclusion at which he arrives from its being couched in the language of the review to which we have referred. Foundling hospitals are mischievous, "because they have no influence on those evils which they were instituted to prevent; because the relief they proffer can be obtained only by a deliberate sacrifice of the best feelings of our nature; because they are liable to abuses, which it is almost impossible to prevent; and because, while they entail a great expense upon the country, they preserve the lives of but a very small proportion of their inmates."*

We know not whether the writer of these judicious conclusions would

* No. XXVI, April, 1842, p. 293.

be disposed to go to the length to which we have indicated our disposition to advance in the matter of public charities; but we are so far disposed to generalize that we would extend our condemnation to all public establishments, which are not in the nature of *prevention*. If any species of public charity ought to be supported by the government, assuredly hospitals and dispensaries are such charities, and yet these have been largely provided by the benevolence of individuals; and though, not by any means free from some of the abuses of foundling hospitals, there can be little doubt that they are much more free from objection, than if they were public institutions supported by the public purse. The real position in life and wants of their inmates is much better known to the subscribers, and those who have the privilege of recommendation, than it could be to government officers; and thus a part, at least, of the abuses to which all charities are liable is guarded against.

We cannot conclude this notice of Dr. Melzer's admirable work, without again reverting to his censure of our countryman Malthus; and we cannot help regarding the conclusion at which he has arrived, respecting the evils of foundling hospitals, as applicable *mutatis mutandis* to all systems of poor-laws, against which our distinguished countryman so earnestly protested.

A short and simple proverb, Prevention better than cure, is at the root of all these questions. "Donnez plus de moralité aux classes ouvrières et il y aura beaucoup moins d'enfants trouvés," (the concluding quotation of Dr. Melzer's work,) admits of extension in many directions. For instance, we would say to England:—Give more intellectual and moral training, and you will have fewer criminals; commit less waste, and you will have fewer paupers; prevent disease and preserve health, and you will need fewer hospitals and workhouses; make the employers of labour responsible for life and limb, and you will have fewer accidents; look to the construction of ships and the education of masters, and you will have fewer shipwrecks; and so on through the length and breadth of your legislation. If this simple proverb will not suffice for your guidance, add this other homely saying, Waste not, want not. If you will not take measures for preventing the refuse of our towns from flowing into the sea, then you must import manure, or corn and meat, or both. There is no other alternative. If you will not consent to that free competition of nation with nation, which, by stimulating all to exertion, makes all men prosperous, then must you take the consequences of your so-called protection,—sloth, ignorance, poverty, revenue laws, preventive service, law-made crimes, and all the waste of time and money which these entail. By your discountenance of foundling hospitals, you show that you are not ignorant of the true principles of political and Christian economy. We earnestly commend the poor-laws to your best attention. You are the richest nation in Europe—you have the largest proportion of paupers. Can this be explained on any other supposition than that they are *law-made* paupers? Try the system of *prevention*: it cannot fail more egregiously than the system of *cure* has done. The present system has the condemnation of the past: its rival has all the promises of the future.

